# Association of sugar-sweetened beverages with executive function in autistic children

**DOI:** 10.3389/fnut.2022.940841

**Published:** 2022-08-22

**Authors:** Shuolin Pan, Xin Wang, Lizi Lin, Jiajie Chen, Xiaoling Zhan, Chengkai Jin, Xiaoxuan Ou, Tingfeng Gu, Jin Jing, Li Cai

**Affiliations:** ^1^Department of Maternal and Child Health, School of Public Health, Sun Yat-sen University, Guangzhou, China; ^2^Key Laboratory of Brain, Cognition and Education Sciences, Ministry of Education; Institute for Brain Research and Rehabilitation, South China Normal University, Guangzhou, China; ^3^Guangdong Provincial Engineering Technology Research Center of Environmental Pollution and Health Risk Assessment, Department of Occupational and Environmental Health, School of Public Health, Sun Yat-sen University, Guangzhou, China

**Keywords:** sugar-sweetened beverage, children, autism spectrum disorder, executive function, cross-sectional study

## Abstract

The association between sugar-sweetened beverages (SSBs) consumption and executive function (EF) among typically developing (TD) children has been investigated in previous studies but with inconsistent results. Furthermore, this relationship has been less investigated among autistic children who perform worse in EF compared with TD children. In this study, we aimed to investigate the association between SSB consumption and EF in autistic children, and whether the association between SSB and EF in autistic children is different from that in TD children. We recruited 106 autistic children and 207 TD children aged 6–12 years in Guangzhou, China. Children’s EF was assessed by using the Chinese version of parent-reported Behavior Rating Inventory of Executive Function, Stroop Color–Word Test, and working memory subscales of the Chinese version of Wechsler Intelligence Scale for children, Fourth edition. Meanwhile, we assessed children’s dietary intake and SSB consumption with a validated Food Frequency Questionnaire. In this study, 70 (66.0%) autistic children consumed SSB and 20 (18.9%) of them consumed more than two servings SSB a week. Among autistic children, over two servings per week SSB consumption was associated with poorer performance in emotional control [β = 7.20, 95% confidence interval (CI): 0.94–13.46] and plan/Organize (β = 6.45, 95% CI: 0.27–12.63). The association between over two servings/week SSB consumption and emotional control among autistic children was significantly different from that among TD children (β_*ASD*_ = 7.20; β_*TD*_ = −3.09, *Z* = 2.72, *p* = 0.006). Results of this study show that SSB consumption was associated with an impairment in some subscales of EF in autistic children. Furthermore, the association between SSB and EF in autistic children might be different from that in TD children.

## Introduction

Consumption of sugar-sweetened beverages (SSBs) among children remains at a high level, especially in low- and middle-income countries, including China (1–3). SSBs such as carbonated beverages and sugar-sweetened fruit juice beverages, were suggested to have an association with an increased risk of children’s physical health problems, including dental caries, obesity(s) and other metabolic diseases (4, 5). Some previous studies suggested that SSB consumption was linked with mental problems, such as impaired cognitive function (6, 7), executive function (EF) (8), as well as behavioral problems, including hyperactivity problems and emotional symptoms (9, 10).

Executive function is an umbrella term for functions that include two dimensions and associate with a child’s cognitive functioning, behavior, emotional control, and social interaction (11). Autism spectrum disorder (ASD) is a heterogeneous neurodevelopmental disorder with impaired social communication and interaction, repetitive behaviors, and varying levels of intellectual disability (12). Compared with typically developing (TD) children, children with ASD performed significantly worse in EF (13–16). Although EF impairment commonly exists in autistic children, high-order EF will have sustainable development and is sensitive to environmental factors [e.g., nutrition (17), air pollution (18), lifestyle (19, 20), and socioeconomic status (21)] during school age. Therefore, it is of significance to improve EF or reduce impairment of EF in autistic children by controlling some environmental factors, especially the highly modifiable dietary factors including SSB.

Studies in healthy animals have shown that fructose consumption could adversely influence synaptic plasticity and cognition (22–28). In population studies, previous reports have investigated the association of SSB consumption with EF among school-aged TD children, but with inconsistent findings. Some studies indicated that SSB had an inverse relationship with all the indexes of EF (8, 9, 29–32), but a few of them indicated that SSB had no relationship with some of the indexes or could be beneficial for children’s cognition EF (29, 33, 34). Inconsistencies in the findings could be partly explained by difference in assessments of EF, ethnicity, and economic development levels.

Although previous studies indicated that SSB consumption was inversely related with EF in school-aged TD children, few studies investigated the relationship of SSB with EF in autistic children (35, 36). Furthermore, several studies suggested that autistic children had a high preference for energy-dense foods, leading to a high consumption of sugar, juice, and sweetened beverages (37–41). Since the food preference and EF of autistic children was different from TD children, it is reasonable to hypothesize that the association between SSB and EF in autistic children may be different from that in TD children. Therefore, we aimed to investigate (1) the association between SSB consumption and EF of autistic children; and (2) whether the association between SSB and EF in autistic children is different from that in TD children.

## Materials and methods

### Study design and participants

In this cross-sectional study, a total of 107 autistic children and 209 TD children aged 6–12 years were recruited from a study entitled “the Guangzhou Longitudinal Study of Children with ASD” in Guangzhou, China. The autistic children had to have a historical diagnosis of ASD, autism, or Asperger’s syndrome and be confirmed by two professional child psychiatrists (Xiuhong Li and Jin Jing) according to the Diagnostic and Statistical Manual of Mental Disorders, Fifth Revision (DSM-5) criteria.

Both groups conformed to the additional inclusion criteria as follows: (1) chronological age between 6 years 0 month and 12 years 11 months 30 days; (2) voluntarily participation of the children’s parents; (3) without known genetic or chromosomal abnormalities or severe visual or hearing impairment; and (4) without any other medical diagnosis of neuropsychiatric disorders such as ADHD, seizures, Tourette syndrome, head trauma, cerebral palsy, or other movement disorders that would interfere with study assessment.

In the current study, one autistic child had missed data from subscales score of the Behavior Rating Inventory of Executive Function (BRIEF) and two TD children had an Intelligence Quotient (IQ) below 70. They were not included in any of the analysis. A subsample of 106 autistic children (89 boys and 17 girls) and 207 TD children (113 boys and 94 girls) were included for final analysis. All 313 participants took part in the Stroop Color–Word Test (SCWT). Because of its presupposition of literacy, only 76 autistic children and 173 TD children finished SCWT. At the research center, 99 autistic children and 207 TD children finishing the IQ test. They had complete scores of four index scores and a full score of IQ (the flowchart was in Supplementary Figure 1).

### Procedure

Children underwent face-to-face measures performed by well-trained psychologists and research assistants at the research center. All the parents of the participants were provided with written consent. This study was approved by the Ethical Review Committee for Biomedical Research at Sun Yat-sen University (2015-No.29).

### Measures

#### Assessment of executive function

We evaluated EF of children *via* parent-reported questionnaire and behavioral experiment.

##### Behavior Rating Inventory of Executive Function

Behavior Rating Inventory of Executive Function is a parent-reported questionnaire for parents of children aged 6–18 years. Parents were asked to rate the 86 items by evaluating how often the problem bothered their child in the past 6 months. The 86 items are rated into three ranks, which are “never,” “sometimes,” and “often,” corresponding to the scores “1,” “2,” and “3,” respectively. The BRIEF comprises three composite indexes (i.e., behavioral regulation index, BRI; metacognition index, MI; and global executive composite, GEC). The BRI reflects the ability to shift cognitive set and modulate emotions and behaviors *via* appropriate inhibitory control, containing three subscales (i.e., inhibit, shift, and emotional control). The MI reflects the ability to initiate, plan, organize, and sustain future oriented problem-solving in working memory, containing initiate, working memory, plan/organize, organization, and monitor subscales. The GEC represents a sum of all the eight subscale scores. The BRI, MI, and GEC were converted into T-scores (mean = 50, standard deviation = 10) and standardized by gender and age. Higher scores indicate greater impairment in EF. Among the school-aged children in China, the subscales and total scores have good internal consistency (0.74–0.96), except for the initial subscale (0.61), and good test-retest reliability (0.68–0.89) (42).

##### Stroop Color–Word Test

The SCWT is a widely used neuropsychological measure that can assess inhibitory control (43). Due to its presupposition of literacy, the test is limited to school-aged children. In the current study, this test consists of three subtasks, namely, reading names of colors serially, naming the colors, and naming the color of ink instead of the words. Each subtask contains 10 trials, and each trail presents one kind of color or a color word. The E-Prime 2.0 on computer was used to run the task and record the response. The participants were asked to respond by pressing buttons on the keyboard as quickly and accurately as possible. The average reaction times (RTs) of all the correct answers would replace the RT of all the wrong answers. Stroop interference (SI) is the difference in RTs between naming the color and naming the color of the ink instead of the words. We recorded the correct rate of Stroop and SI to assess the participants’ performance on SCWT.

##### Working Memory Index

We also used Working Memory Index (WMI) in the Chinese version of Wechsler Intelligence Scale for Children, Fourth Edition (WISC-IV) to assess children’s EF (44). WISC-IV has an internal consistency ranging from 0.98 to 0.99 and test-retest reliability ranging from 0.71 to 0.86. The WMI is made up of Digit Span (DS) and Letter-Number Sequencing (LNS) subtests. DS includes Digit Span Forward (DSF) and Digit Span Backward (DSB), and scoring combines the total number of correctly repeated digit strings. The LNS subtests require children to repeat the sequence of letters and numbers provided randomly in a predetermined order. An arithmetic subtest may be used as a replacement if a participant cannot finish one of the two aforementioned subtests. In this subtest, children are required to answer verbally presented arithmetic problems. A higher score of WMI indicates greater working memory ability.

##### Dietary intake and sugar-sweetened beverage consumption

Dietary intake was assessed by using a validated Food Frequency Questionnaire (FFQ) (45). Parents of children were required to report the frequency and amount of food their children consumed during the past 7 days. The list of foods included cereals, vegetables, fruits, dairy foods, soybeans, red meat and products, poultry and game, fish and shrimp, nuts, eggs, salt, SSBs, cooking oil, and water. To assist the interview, we provided food photographs with standard portion sizes.

For SSB consumption, parents were asked to answer the questions “During the past seven days, how many times had your child drunk SSB (e.g., Coca-Cola, Sprite, orange juice, etc.)?” and “How many glasses (250 milliliters per glass) of SSB had your child consumed on average each time?” In this study, we used the term “servings” to describe the intake of SSB, and 250 ml of SSB was defined as one serving. We also classified the SSB servings per week into three categories as “0 servings/week, >0–2 servings/week, and >2 servings/week.”

### Assessment of covariates

#### Social demography factors

Demographic information such as children’s gender, age, maternal and paternal education level, and per capita monthly household income was obtained *via* questionnaires.

#### Physical activity and sedentary time

Physical activity (PA) and sedentary time (ST) during the past 7 days were assessed *via* the International Physical Activity Questionnaire Short Form (IPAQ-SF). Parents were required to report the weekly frequency and duration of vigorous-intensity activities (VPA), moderate activities (MPA), and walking. ST includes after-school homework time and screen time of the participants (including watching television and taking online courses). Three levels of PA were classified as “HEPA active,” “Minimally active,” and “Inactive” according to the criteria based on different energy requirements of activities (46). We also classified the screen time per day into two categories as “<2 hours/day” and “≥2 hours/day.”

#### Anthropometric measurements

Anthropometric measurements were taken according to the standard protocol of the National Standard Test Method for Students’ Physical Health. The body mass index (BMI) of each participant was calculated as weight (kg) divided by height squared (m^2^). According to the Chinese Standards, we classified the BMI of the children into four categories, namely, underweight, healthy weight, overweight, and obese (47, 48).

### Statistical analyses

Means and standard deviations were calculated to describe continuous variables and percentages to describe categorical variables. We compared demographic information between children with ASD and TD children using *t*-tests and Chi-square tests.

We used generalized linear models to investigate the associations of EF and SSB consumption. Crude models were fitted without any adjustments. Adjusted model 1 was fitted by adjusting the child’s age, gender, maternal and paternal education level, and per capita monthly household. Adjusted model 2 was fitted by further adjusting screen-exposure time, category of PA, category of BMI, and water intake. Adjusted model 3 further adjusted the category of IQ (<70 and ≥70). To investigate whether the association between SSB and EF of autistic children is different from that of TD children, we further implemented a test for coefficient estimate (β), based on the point estimate and standard error (SE):


$$Z=\frac{\beta_{A\,S\,D}-\beta_{T\,D}}{\sqrt{s\,e\,{\left(\beta_{A\,S\,D}\right)}^{2}+s\,e\,{\left(\beta_{A\,S\,D}\right)}^{2}}}$$

All statistical analysis was conducted with the R 4.1.3 statistical software (R Core Team) (49). Coefficient estimate (β) with a 95% confidence interval (CI) were presented as the results. A two-sided *p*-value < 0.05 was considered statistically significant.

## Results

### Characteristics of autistic children and typically developing children

A total of 106 autistic children and 207 TD children aged 6–10 years participated in the investigation (Table 1). Among autistic children, the majority of children (84.0%) were boys. Among TD children, approximately half of the children were boys (54.6%).

**TABLE 1:** Demographic characteristics of children with ASD and TD children.

	ASD (*N* = 106)	TD (*N* = 207)	*P-value*
	*N* (%)/mean (SD)	*N* (%)/mean (SD)	
**Age (years)**	7.7 (1.3)	7.8 (1.3)	0.854
**Gender**			<0.001***
Boys	89 (84.0)	113 (54.6)	
Girls	17 (16.0)	94 (45.4)	
BMI	16.6 (2.6)	15.8 (2.4)	0.007**
**BMI category**			0.060
Underweight	10 (9.4)	26 (12.6)	
Healthy weight	68 (64.2)	149 (71.9)	
Overweight	12 (11.3)	19 (9.2)	
Obese	16 (15.1)	13 (6.3)	
**Physical activity category**			0.009**
Inactive	11 (10.4)	7 (3.4)	
Minimally active	46 (43.4)	75 (36.2)	
HEPA active	49 (46.2)	125 (60.4)	
**Maternal education**			0.002**
High school degree or less	44 (41.5)	52 (24.9)	
Bachelor’s degree or above	62 (58.5)	157 (75.1)	
**Paternal education**			0.730
High school degree or less	40 (37.7)	74 (35.7)	
Bachelor’s degree or above	66 (62.3)	133 (64.3)	
**Per capita monthly household income**			<0.001***
<¥8000	61 (57.5)	52 (25.1)	
≥¥8000	45 (42.5)	155 (74.9)	

ASD, autism spectrum disorder; TD, typically developing; SD, standard deviation; BMI, body mass index.

**p < 0.01; ***p < 0.001.

Autistic children had a significantly higher average BMI (16.6 ± 2.6 vs. 15.8 ± 2.4; *p* < 0.01) compared with TD children. Autistic children showed higher rates of obesity (15.1 vs. 6.3%) and lower rates of healthy weight (64.2 vs. 71.9%). The PA category also had a significant difference between children with ASD and TD children (inactive: 10.4 vs. 3.4%; minimally active: 43.4 vs. 36.2%; HEPA active: 46.2 vs. 60.4%; *p* < 0.01). Lower maternal education level and lower monthly household income (both *p* < 0.01) were noted in children with ASD.

### Comparison of sugar-sweetened beverage in children with autism spectrum disorder and typically developing children

**FIGURE 1:**
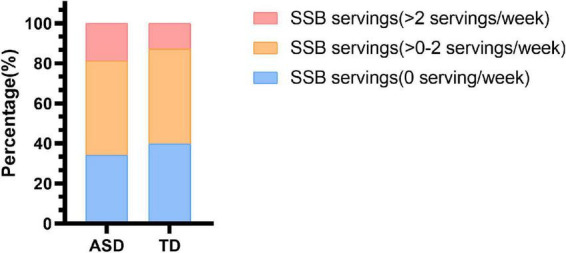
Sugar-sweetened beverage consumption in children with ASD and TD children. SSB, sugar-sweetened beverage; ASD, autism spectrum disorder; TD, typically developing.

The mean (SD) of servings of SSB consumption per week was 1.3 (1.6) among autistic children and 1.2 (1.7) among TD children. A total of 20 (18.9%) autistic children and 26 (12.6%) TD children reported consuming no less than two servings of SSB per week. Thirty-six (34.0%) autistic children and 83 (40.1%) TD children reported consuming 0 servings of SSB per week (Figure 1). However, there was no statistically significant difference between the SSB consumption of autistic children and TD children (Supplementary Table 1).

### Executive function of autistic children and typically developing children

**FIGURE 2:**
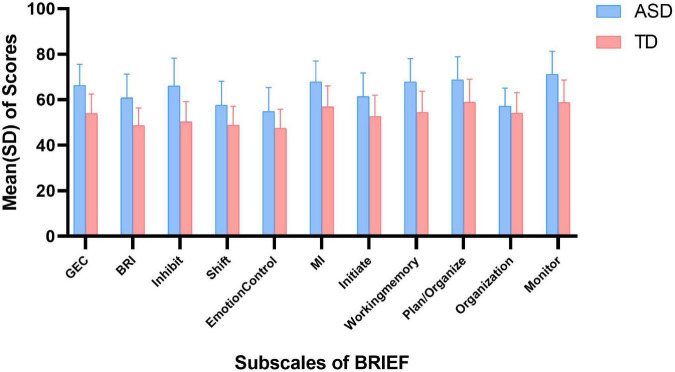
Scores of BRIEF in ASD and TD children. ASD, autism spectrum disorder; TD, typically developing; SD, standard deviation; GEC, global executive component; BRI, behavioral regulation index; MI, metacognition index.

The scores of BRIEF in autistic children and TD children are shown in Figure 2. In parent-report BRIEF, the mean (SD) score of GEC in autistic children was 66.3 (9.2), while that in TD children was 53.9 (8.6). Besides, the mean (SD) of BRI and MI in autistic children was 60.8 (10.5) and 67.9 (9.1), while that in TD children was 48.6 (7.8) and 56.8 (9.3), respectively. In behavioral experiment, the mean (SD) of correct rate of Stroop was 0.9 (0.1) in both the groups. And the mean (SD) of SI and WMI in autistic children was 401.1 (464.3) and 90.1 (18.8), while that in TD children was 407.9 (384.1) and 104.6 (12.5), respectively.

### Association between sugar-sweetened beverage consumption and executive function in autistic children

The association between SSB consumption and EF in autistic children is summarized in Tables 2, 3 and Supplementary Table 2. In model 3, autistic children who consumed more than two servings of SSB per week were associated with significantly higher scores of emotional control (β = 7.20, 95% CI: 0.94 to 13.46, *p* = 0.024) and plan/organize (β = 6.45, 95% CI: 0.27 to 12.63, *p* = 0.041), compared with those who consumed none. The results indicated that autistic children who have more than two servings of SSB per week might have worse performance in emotional control and plan/organize than those who did not drink SSB. There was no significant association between SSB consumption and EF among TD children (Tables 2, 3 and Supplementary Table 3).

**TABLE 2:** Association between SSB consumption and scores of BRIEF among the two groups, and the comparison between the coefficient estimate (β) of the two groups.

	Coefficient estimate (β, adjust model 3^α^)				*Z* ^δ^	*^p^* ^ε^
	ASD		TD			
	β_*ASD*_ (95% CI)	*p* ^β^	β_*TD*_ (95% CI)	*p* ^γ^		
**Inhibit**						
0 serving/week	Reference		Reference			
>0–2 servings/week	−0.53 (−6.34, 5.27)	0.857	−0.71 (−3.41, 1.98)	0.604	0.06	0.956
>2 servings/week	3.83 (−2.58, 11.24)	0.311	−3.37 (−7.63, 0.89)	0.121	1.65	0.099
**Shift**						
0 serving/week	Reference		Reference			
>0–2 servings/week	0.46 (−4.55, 5.46)	0.858	−1.32 (−3.79, 1.15)	0.295	0.62	0.533
>2 servings/week	4.43 (−1.97, 10.82)	0.175	−2.83 (−6.74, 1.07)	0.155	1.90	0.057
**Emotional control**						
0 serving/week	Reference		Reference			
>0–2 servings/week	0.91 (−3.99, 5.82)	0.715	−1.00 (−3.49, 1.49)	0.431	0.68	0.496
>2 servings/week	7.20 (0.94, 13.46)	0.024*	−3.09 (−7.03, 0.84)	0.123	2.72	0.006**
**Initiate**						
0 serving/week	Reference		Reference			
>0–2 servings/week	−0.28 (−5.33, 4.78)	0.915	−0.00 (−2.85, 2.85)	0.999	−0.71	0.480
>2 servings/week	−0.27 (−6.72, 6.18)	0.935	3.04 (−1.46, 7.54)	0.185	−1.16	0.245
**Working memory**						
0 serving/week	Reference		Reference			
>0–2 servings/week	0.03 (−4.53, 4.58)	0.991	0.90 (−1.83, 3.62)	0.520	−0.32	0.748
>2 servings/week	3.62 (−2.19, 9.44)	0.222	−0.41 (−4.71, 3.89)	0.852	1.09	0.276
**Plan/organize**						
0 serving/week	Reference		Reference			
>0–2 servings/week	1.46 (−3.37, 6.30)	0.553	1.19 (−1.77, 4.16)	0.431	0.09	0.926
>2 servings/week	6.45 (0.27, 12.63)	0.041*	2.37 (−2.31, 7.06)	0.321	1.03	0.302
**Organization**						
0 serving/week	Reference		Reference			
>0–2 servings/week	−1.25 (−5.15, 2.65)	0.530	1.05 (−1.68, 3.77)	0.452	−0.95	0.343
>2 servings/week	0.88 (−4.10, 5.87)	0.728	−0.09 (−4.39, 4.21)	0.967	0.29	0.772
**Monitor**						
0 serving/week	Reference		Reference			
>0–2 servings/week	0.77 (−4.25, 5.79)	0.764	−0.73 (−3.66, 2.21)	0.628	0.51	0.613
>2 servings/week	2.56 (−3.85, 8.97)	0.434	−2.92 (−7.55, 1.71)	0.216	1.35	0.174
**BRI**						
0 serving/week	Reference		Reference			
>0–2 servings/week	0.28 (−4.63, 5.19)	0.911	−1.00 (−3.34, 1.34)	0.401	0.46	0.644
>2 servings/week	6.04 (−0.22, 12.31)	0.059	−3.53 (−7.22, 0.16)	0.061	2.58	0.010*
**MI**						
0 serving/week	Reference		Reference			
>0–2 servings/week	0.25 (−4.12, 4.61)	0.912	0.63 (−2.13, 3.40)	0.653	−0.14	0.885
>2 servings/week	3.50 (−2.06, 9.07)	0.218	0.64 (−3.73, 5.00)	0.775	0.79	0.438
**GEC**						
0 serving/week	Reference		Reference			
>0–2 servings/week	0.35 (−3.97, 4.66)	0.875	0.02 (−2.54, 2.58)	0.989	0.13	0.897
>2 servings/week	4.85 (−0.66, 10.35)	0.084	−0.86 (−4.86, 3.33)	0.691	1.64	0.101

^α^Adjusted for age, sex, maternal education, paternal education, family income, screen time category, physical activity category, BMI category water, and IQ.

^β^p-Value of the correlation between SSB consumption and BRIEF in autistic children.

^γ^p-Value of the correlation between SSB consumption and BRIEF in TD children.

${}^{\delta }\,Z=\frac{\beta_{A\,S\,D}-\beta_{T\,D}}{\sqrt{s\,e\,{\left(\beta_{A\,S\,D}\right)}^{2}+s\,e\,{\left(\beta_{A\,S\,D}\right)}^{2}}}$.

^ε^P value of the comparison between the β_ASD_ and β_TD_.

*p < 0.05; **p < 0.01.

**TABLE 3:** Association between SSB consumption and behavioral experiments among the two groups, and the comparison between the β of the two groups.

	Coefficient estimate (β, adjust model 3^α^)				*Z* ^δ^	*p* ^ε^
	ASD		TD			
	β _*ASD*_ (95% CI)	*p* ^β^	β _*TD*_ (95% CI)	*p* ^γ^		
**Correct rate of Stroop**						
0 serving/week	Reference		Reference			
>0–2 servings/week	−0.02 (−0.08, 0.05)	0.593	0.01 (−0.03, 0.04)	0.703	−0.83	0.405
>2 servings/week	0.00 (−0.08, 0.09)	0.954	0.02 (−0.03, 0.08)	0.426	−0.40	0.689
**SI**						
0 serving/week	Reference		Reference			
>0–2 servings/week	−13.11 (−282.12, 255.90)	0.924	−65.50 (−194.84, 63.83)	0.321	0.34	0.731
>2 servings/week	−148.01 (−502.67, 206.65)	0.413	−134.19 (−336.36, 67.99)	0.193	−0.07	0.947
**WMI**						
0 serving/week	Reference		Reference			
>0–2 servings/week	2.41 (−4.83, 9.65)	0.514	−1.06 (−3.74, 1.62)	0.439	0.88	0.378
>2 servings/week	−3.82 (−13.17, 5.53)	0.423	−1.41 (−5.65, 2.82)	0.513	−0.46	0.645

^α^Adjusted for age, sex, maternal education, paternal education, family income, screen time category, physical activity category, BMI category water, and IQ.

^β^p-Value of the correlation between SSB consumption and BRIEF in autistic children.

^γ^p-Value of the correlation between SSB consumption and BRIEF in TD children.

${}^{\delta }\,Z=\frac{\beta_{A\,S\,D}-\beta_{T\,D}}{\sqrt{s\,e\,{\left(\beta_{A\,S\,D}\right)}^{2}+s\,e\,{\left(\beta_{A\,S\,D}\right)}^{2}}}$.

^ε^p-Value of the comparison between the β_ASD_ and β_TD_.

### Comparison of the correlation between sugar-sweetened beverage consumption and executive function in two groups

We further investigated the difference between the correlation (β) between SSB and EF in the two groups (Tables 2, 3). The relationship between >2 servings/week SSB consumption and emotional control in autistic children significantly differed from that in TD children (*Z* = 2.72, *p* = 0.006). And the correlation between >2 servings/week SSB consumption and BRI was significantly different from that in TD children (*Z* = 2.58, *p* = 0.010). Besides, the correlation between SSB consumption and other subscales of BRIEF in autistic children, as well as the correlation between SSB consumption and their behavioral experiment results had no significant difference compared with that in TD children.

## Discussion

In the current study, about two-thirds of autistic children consumed SSB. About one in five autistic children consumed an average of two servings of SSB per week. Higher SSB consumption was associated with worse performance on the BRIEF subscale of emotional control and plan/organize in autistic children. In addition, we observed that the relationship between >2 servings/week SSB consumption and EF in autistic children was different from that in TD children. However, we did not find significant associations of SSB consumption with inhibition-control ability measured by the SCWT and WMI measured by WISC-IV test.

Some of our findings were consistent with previous studies among TD children. Most of the previous studies investigated the association between consumption of SSB and EF in TD children (6–8, 29). A randomized, cross-over study of 29 school-aged children assessed children’s EF by using a selection of tests from the Cognitive Drug Research (CDR) computerized assessment system (29). This study reported that the glucose drink was associated with great declines in attention and episodic secondary memory but had no association with working memory. In a cross-sectional analysis of over 6,000 children in Guangzhou, higher SSB consumption was associated with poorer performance in all the subscales and composite scores of parent-reported BRIEF (8). In addition, a cohort study of 1,234 children in the United States used Kaufman Brief Intelligence Test (KBIT-II) to assess verbal and non-verbal global intelligence and the Wide Range Assessment of Memory and Learning for visual memory (7). The results revealed that additional SSB consumption in early childhood was associated with poorer verbal intelligence at mid-childhood. These studies all indicated that SSB consumption had an association with impairment of EF in children. The findings may vary in the indexes or strength of the association due to different assessment of EF, adjusted covariates, study population, and methods in quantifying SSB consumption.

Although the previous studies indicated that SSB consumption was inversely related with EF in school-aged TD children, few studies investigated the relationship of SSB with EF in autistic children. In our study, we focused on autistic children in China. We assessed children’s EF with the SCWT, WMI of WISC-IV, and the Chinese version of the parent-reported BRIEF. Besides social demography and economic factors, we also adjusted for the screen time category, water intake, and IQ category, which had a relationship with children’s EF or SSB consumption (50–52). After adjustment for the covariates, our findings showed that having more than two servings of SSB per week was associated with increasing scores of emotional control and plan/organize in autistic children. Emotional control subscale describes children’s ability to modulate emotional responses appropriately. Plan/organize subscale describes the ability to anticipate future events, set goals, and understand and communicate key concepts. This finding indicated that in autistic children, a higher level of SSB consumption might have an inverse association with performance on appropriately modulating emotional responses and anticipating future events. Findings of our study add to the literature on the adverse associations of SSB consumption and EF in autistic children. More studies from different regions are needed to confirm this relationship in this population. In addition, the association between emotional control and SSB consumption in autistic children was significantly different from that in TD children. Some previous studies indicated that sensory sensitivity was commonly found in autistic population (12, 53). This kind of sensory sensitivity was associated with autistic person’s intolerance of uncertainty and subsequent anxiety (53). In this study, autistic children might be more sensitive to a higher level of SSB consumption and performed worse in emotional control and BRI. The BRI also reflects the ability to modulate emotions. This finding indicated that autistic children’s emotional control ability might be more susceptible to high SSB consumption than that of TD children.

According to previous studies, the biological mechanism underlying the relationship between SSB consumption and EF is plausible. Most of these studies proposed that a high level of sucrose or fructose would increase inflammation and oxidative stress and decrease neurotrophins, which may be one of the pathways to explain the relationship. Animal evidence from general rat models indicated that 4 weeks of sucrose–fructose drinks feeding would induce increased expression of pro-inflammatory mediator genes such as IL-1β and IL-6 (22, 28). In the hypothalamus of sucrose–fructose fed rats, Toll-like receptor 4 (TLR4) as well as nuclear factor kappa-light-chain-enhancer of activated B cells (NF-κB) have higher levels than the control group. As for autism, many studies indicated that levels of brain cytokines, including IL-6 and IL-1β, were significantly higher in autistic children compared with TD controls (54–59). Some studies demonstrated that brain IL-6 could mediate autism-like behaviors including heightened anxiety and deficits (60, 61). In an animal study, the researchers developed a mouse model that overexpresses IL-6 in the brain, and they discovered that the elevation of IL-6 in the mouse brain could produce autistic features, including impaired cognitive abilities, deficits in learning, decreased social interactions, as well as abnormal anxiety-like traits and habituation (60). Another study in 223 pre-school aged children indicated that stereotypical behavior and impaired social interactions in autistic children were more pronounced, as certain cytokine (IL-1β, IL-6, and IL-8) levels increased (62). Therefore, SSB consumption may further elevate the level of autistic children’s brain IL-6 and cause severer EF impairment. Compared with TD children, autistic children might be more sensitive to increased brain IL-6 level because of the original IL-6 dysregulation (62, 63). In addition, several studies indicated that IL-6 was associated with emotional problems (64–70). Many studies found elevated IL-6 levels and its membrane-bound receptors in depressed individuals (67–70). Therefore, we might be able to speculate that sucrose–fructose drinks could impair children’s EF by increasing the levels of brain IL-6 and IL-1β and this impairment would be greater in autistic children.

In this study, there were about one-fifth of autistic children who consumed no less than two servings of SSB per week, which contained about 50 g of sugar. However, children are advised to drink less or no SSB according to the current dietary guideline for Chinese (71) or Americans (72). Therefore, autistic children are suggested to decrease the consumption of SSB and improve adherence to nutrition guidelines. Parents, community, and schools should help these children to achieve better dietary quality. There were some limitations in this study. First, it is not possible to infer the causality of SSB consumption and EF based on the cross-sectional study design. Prospective cohort studies would be needed to confirm the long-term associations. Second, recall bias and information bias were inevitable because SSB consumption, children’s performance on EF, and demographic factors were based on parent-reported questionnaires in our study. However, the face-to-face interview and other quality control method may help us to reduce the recall bias. Third, although we adjusted for many individual confounders in the models, we cannot rule out the effects of other unmeasured potential confounding factors such as parental smoking and parental mental status.

## Conclusion

We found that SSB consumption was inversely related to EF in autistic children, and the emotional control ability of autistic children might be more susceptible to SSB. These findings highlight the necessity of limiting autistic children’s SSB consumption.

## Data availability statement

The generated datasets are available by request to the corresponding author JJ, jingjin@mail.sysu.edu.cn.

## Ethics statement

The studies involving human participants were reviewed and approved by the Ethical Review Committee for Biomedical Research, Sun Yat-sen University (2015-No.29). Written informed consent to participate in this study was provided by the participants’ legal guardian/next of kin.

## Author contributions

LC and JJ: conceptualization, supervision, project administration, and funding acquisition. SP: methodology and formal analysis, and writing—original draft preparation. XW and LC: study design and manuscript revision. SP, XW, JC, XZ, CJ, XO, and TG: data collection. XW: data curation. SP, XW, LL, JC, XZ, CJ, XO, TG, LC, and JJ: writing—review and editing. All authors contributed to the article and approved the submitted version.
