# Supplementary material for: Association of sugar-sweetened beverages with executive function in autistic children
Source: Front Nutr. 2022 Aug 22;9:940841. doi: 10.3389/fnut.2022.940841 (PMC9447427; doi:10.3389/fnut.2022.940841)
Supplement: Supplementary file 1 [file Data_Sheet_1.docx]

Supplementary Material

**Supplementary Table. S1** Comparison of sugar-sweetened beverage, in children with ASD and TD children

|  | ASD (N=106) | TD (N=207) | *P* value |
| --- | --- | --- | --- |
|  | N (%)/Mean (SD) | N (%)/Mean (SD) |  |
| SSB servings per week | 1.3(1.6) | 1.2(1.7) | 0.473 |
| SSB servings per week category |  |  | 0.274 |
| 0 serving/week | 36(34.0) | 83(40.1) |  |
| >0-2 servings/week | 50(47.2) | 98(47.3) |  |
| >2 servings/week | 20(18.9) | 26(12.6) |  |

**Abbreviation:** SSB, sugar-sweetened beverages; ASD, autism spectrum disorder; TD, typically developing; SD, Standard deviation;

**Supplementary Table. S2** Association between SSB servings and EF in children with ASD

| Executive function |  |  | **SSB [***β* (95% confidence interval)] | | |  |
| --- | --- | --- | --- | --- | --- | --- |
|  | Crude model ^a^ | *P* value | Adjusted model 1^β^ | *P* value | Adjusted model 2^γ^ | *P* value |
| Inhibit |  |  |  |  |  |  |
| 0 serving/week | Reference |  | Reference |  | Reference |  |
| >0-2 servings/week | -2.18(-7.44,3.07) | 0.416 | -2.20(-7.65,3.25) | 0.431 | -1.65(-7.56,4.25) | 0.583 |
| >2 servings/week | 2.17(-4.54,8.87) | 0.527 | 2.27(-4.68,9.22) | 0.523 | 3.07(-4.54,10.67) | 0.429 |
| Shift |  |  |  |  |  |  |
| 0 serving/week | Reference |  | Reference |  | Reference |  |
| >0-2 servings/week | -0.11(-4.61,4.38) | 0.961 | -0.50(-5.12,4.12) | 0.832 | 0.34(-4.59,5.27) | 0.893 |
| >2 servings/week | 4.47(-1.27,10.20) | 0.127 | 4.96(-0.92,10.85) | 0.102 | 4.25(-1.99,10.69) | 0.179 |
| Emotional control |  |  |  |  |  |  |
| 0 serving/week | Reference |  | Reference |  | Reference |  |
| >0-2 servings/week | -0.92(-5.37,3.54) | 0.686 | -0.64(-5.16,3.88) | 0.783 | 0.76(-4.07,5.59) | 0.757 |
| >2 servings/week | 5.76(0.08,11.45) | **0.047*** | 6.28(0.52,12.04) | **0.033*** | 7.09(0.88,13.31) | **0.025*** |
| Initiate |  |  |  |  |  |  |
| 0 serving/week | Reference |  | Reference |  | Reference |  |
| >0-2 servings/week | -1.26(-5.75,3.22) | 0.581 | -1.67(-6.34,3.00) | 0.483 | -1.15(-6.26,3.96) | 0.659 |
| >2 servings/week | 0.21(-5.52,5.93) | 0.944 | 0.10(-5.85,6.06) | 0.974 | -0.87(-7.44,5.71) | 0.797 |
| Working memory |  |  |  |  |  |  |
| 0 serving/week | Reference |  | Reference |  | Reference |  |
| >0-2 servings/week | -0.59(-4.94,3.76) | 0.790 | -0.63(-5.13,3.88) | 0.785 | -1.11(-5.85,3.63) | 0.647 |
| >2 servings/week | 2.84(-2.71,8.39) | 0.316 | 2.97(-2.77,8.72) | 0.310 | 2.85(-3.25,8.95) | 0.359 |
| Plan/organize |  |  |  |  |  |  |
| 0 serving/week | Reference |  | Reference |  | Reference |  |
| >0-2 servings/week | 0.77(-3.54,5.08) | 0.727 | 0.64(-3.85,5.14) | 0.779 | 1.00(-2.83,6.73) | 0.426 |
| >2 servings/week | 6.34(0.84,11.84) | **0.024*** | 6.51(0.78,12.24) | **0.026*** | 6.13 (-0.04,12.31) | 0.052 |
| Organization of materials |  |  |  |  |  |  |
| 0 serving/week | Reference |  | Reference |  | Reference |  |
| >0-2 servings/week | -1.68(-5.13,1.77) | 0.340 | -1.67(-5.21,1.88) | 0.356 | -1.29(-5.13,2.55) | 0.511 |
| >2 servings/week | 0.39(-4.02,4.79) | 0.863 | 0.20(-4.32,4.72) | 0.930 | 0.86(-4.08,5.80) | 0.733 |
| Monitor |  |  |  |  |  |  |
| 0 serving/week | Reference |  | Reference |  | Reference |  |
| >0-2 servings/week | -0.34(-4.76,4.08) | 0.882 | -0.47(-5.07,4.12) | 0.840 | 0.18(-4.82,5.19) | 0.943 |
| >2 servings/week | 1.29(-4.35,6.93) | 0.654 | 1.72(-4.15,7.58) | 0.566 | 2.16(-4.27,8.60) | 0.510 |
| BRI |  |  |  |  |  |  |
| 0 serving/week | Reference |  | Reference |  | Reference |  |
| >0-2 servings/week | -1.37(-5.82,3.07) | 0.546 | -1.37(-5.91,3.18) | 0.557 | -0.28(-5.16,4.61) | 0.912 |
| >2 servings/week | 4.69(-0.98,10.36) | 0.105 | 5.14(-0.66,10.93) | 0.086 | 5.66(-0.62,11.95) | 0.077 |
| MI |  |  |  |  |  |  |
| 0 serving/week | Reference |  | Reference |  | Reference |  |
| >0-2 servings/week | -0.59(-4.48,3.29) | 0.766 | -0.78(-4.83,3.28) | 0.708 | -0.48(-4.88,3.92) | 0.831 |
| >2 servings/week | 2.99(-1.97,7.95) | 0.237 | 3.10(-2.08,8.27) | 0.241 | 3.01(-2.65,8.68) | 0.298 |
| GEC |  |  |  |  |  |  |
| 0 serving/week | Reference |  | Reference |  | Reference |  |
| >0-2 servings/week | -0.90(-4.79,2.99) | 0.649 | -1.03(-5.07,3.00) | 0.616 | -0.38(-4.74,3.98) | 0.864 |
| >2 servings/week | 3.96(-1.01,8.92) | 0.118 | 4.21(-0.94,9.35) | 0.109 | 4.26(-1.25,9.96) | 0.128 |
| Correct rate of Stroop |  |  |  |  |  |  |
| 0 serving/week | Reference |  | Reference |  | Reference |  |
| >0-2 servings/week | -0.01(-0.06,0.05) | 0.844 | -0.01(-0.06,0.05) | 0.832 | -0.02(-0.08,0.05) | 0.563 |
| >2 servings/week | 0.02(-0.06,0.09) | 0.638 | 0.02(-0.05,0.10) | 0.586 | 0.00(-0.08,0.09) | 0.913 |
| SI |  |  |  |  |  |  |
| 0 serving/week | Reference |  | Reference |  | Reference |  |
| >0-2 servings/week | -26.76(-271.42,217.90) | 0.830 | -1.94(-256.35,252.46) | 0.988 | -2.37(-279.19,274.45) | 0.987 |
| >2 servings/week | -46.80(-361.92,268.33) | 0.771 | -50.39(-375.95,275.17) | 0.762 | -162.18(-527.14,202.77) | 0.384 |
| WMI |  |  |  |  |  |  |
| 0 serving/week | Reference |  | Reference |  | Reference |  |
| >0-2 servings/week | -0.24 (-8.46,7.99) | 0.955 | 1.75(-5.88,9.39) | 0.652 | 2.63(-5.82,11.07) | 0.542 |
| >2 servings/week | -4.86(-15.32,5.57) | 0.360 | -5.65(-15.38,4.09) | 0.256 | -5.60(-16.48,5.28) | 0.313 |

**Abbreviation:** SSB, sugar-sweetened beverages; GEC, Global Executive Component; BRI, Behavioral Regulation Index; MI, Metacognition Index; SI, Stroop interference; WMI, Working Memory Index

^a^ Crude model: without adjustment;

^β^ Adjusted model 1, adjusted for age, sex; maternal education; paternal education; family income;

^γ^ Adjusted model 2, further adjusted for screen time, physical activity, BMI category and water;

* *P* < 0.05**Supplementary Table. S3** Association between SSB servings and executive function in children with TD

| Executive function |  |  | **SSB** [*β*(95% confidence interval)] | | |  |
| --- | --- | --- | --- | --- | --- | --- |
|  | Crude model ^a^ | *P* value | Adjusted model 1 ^β^ | *P* value | Adjusted model 2^γ^ | *P* value |
| Inhibit |  |  |  |  |  |  |
| 0 serving/week | Reference |  | Reference |  | Reference |  |
| >0-2 servings/week | -0.64(-3.28,2.00) | 0.635 | -0.30(-2.97,2.38) | 0.829 | -0.57(-3.30,2.16) | 0.680 |
| >2 servings/week | -2.28(-6.26,1.69) | 0.262 | -2.12(-6.23,1.98) | 0.310 | -2.98(-7.28,1.33) | 0.175 |
| Shift |  |  |  |  |  |  |
| 0 serving/week | Reference |  | Reference |  | Reference |  |
| >0-2 servings/week | -1.34 (-3.73,1.05) | 0.271 | -1.09(-3.53,1.35) | 0.383 | -1.19(-3.69,1.31) | 0.349 |
| >2 servings/week | -2.18(-5.78,1.42) | 0.236 | -1.88(-5.62,1.86) | 0.325 | -2.48(-6.42,1.46) | 0.218 |
| Emotional Control |  |  |  |  |  |  |
| 0 serving/week | Reference |  | Reference |  | Reference |  |
| >0-2 servings/week | -1.06 (-3.47,1.34) | 0.386 | -0.79(-3.24,1.66) | 0.527 | -0.88(-3.39,1.64) | 0.494 |
| >2 servings/week | -3.11(-6.74,0.51) | 0.092 | -2.58(-6.34,1.17) | 0.177 | -2.75(-6.72,1.21) | 0.173 |
| Initiate |  |  |  |  |  |  |
| 0 serving/week | Reference |  | Reference |  | Reference |  |
| >0-2 servings/week | 0.17(-2.57,2.91) | 0.904 | 0.29(-2.52,3.10) | 0.841 | 0.10(-2.67,2.96) | 0.946 |
| >2 servings/week | 4.15(0.02,8.28) | **0.049*** | 4.46(0.15,8.77) | **0.043*** | 3.33(-1.18,7.83) | 0.148 |
| Working Memory |  |  |  |  |  |  |
| 0 serving/week | Reference |  | Reference |  | Reference |  |
| >0-2 servings/week | 1.38 (-1.36,4.13) | 0.324 | 1.42(-1.39,4.23) | 0.322 | 1.16(-1.70,4.02) | 0.427 |
| >2 servings/week | 1.13(-3.00,5.26) | 0.592 | 1.48(-2.83,5.78) | 0.502 | 0.32(-4.18,4.83) | 0.889 |
| Plan/organize |  |  |  |  |  |  |
| 0 serving/week | Reference |  | Reference |  | Reference |  |
| >0-2 servings/week | 1.72(-1.24,4.67) | 0.256 | 1.75(-1.25,4.74) | 0.253 | 1.38(-1.64,4.41) | 0.370 |
| >2 servings/week | 3.87(-0.59,8.33) | 0.089 | 4.58(-0.01,9.17) | 0.051 | 2.91(-1.86,7.68) | 0.232 |
| Organization of materials |  |  |  |  |  |  |
| 0 serving/week | Reference |  | Reference |  | Reference |  |
| >0-2 servings/week | 1.62(-1.02,4.26) | 0.229 | 1.36(-1.31,4.03) | 0.317 | 1.14(-1.59,3.87) | 0.413 |
| >2 servings/week | 1.61(-2.37,5.58) | 0.428 | 0.97(-3.12,5.06) | 0.643 | 0.18(-4.13,4.48) | 0.936 |
| Monitor |  |  |  |  |  |  |
| 0 serving/week | Reference |  | Reference |  | Reference |  |
| >0-2 servings/week | -0.67(-3.59,2.24) | 0.651 | -0.33(-3.27,2.62) | 0.828 | -0.54(-3.53,2.46) | 0.726 |
| >2 servings/week | -1.65(-6.04,2.75) | 0.463 | -0.97(-5.49,3.54) | 0.672 | -2.39(-7.11,2.33) | 0.321 |
| BRI |  |  |  |  |  |  |
| 0 serving/week | Reference |  | Reference |  | Reference |  |
| >0-2 servings/week | -1.06(-3.35,1.23) | 0.365 | -0.67(-3.00,1.65) | 0.570 | -0.85 (-3.23,1.53) | 0.483 |
| >2 servings/week | -2.95(-6.39,0.50) | 0.094 | -2.52(-6.08,1.05) | 0.166 | -3.11(-6.87,0.64) | 0.104 |
| MI |  |  |  |  |  |  |
| 0 serving/week | Reference |  | Reference |  | Reference |  |
| >0-2 servings/week | 1.09(-1.65,3.82) | 0.435 | 1.15(-1.65,3.95) | 0.420 | 0.83(-2.00,3.67) | 0.564 |
| >2 servings/week | 2.36 (-1.76,6.48) | 0.262 | 2.68(-1.61,6.97) | 0.221 | 1.20(-3.28,5.67) | 0.600 |
| GEC |  |  |  |  |  |  |
| 0 serving/week | Reference |  | Reference |  | Reference |  |
| >0-2 servings/week | 0.27(-2.25,2.80) | 0.832 | 0.47(-2.11,3.05) | 0.720 | 0.21(-2.42,2.83) | 0.877 |
| >2 servings/week | 0.48(-3.32,4.29) | 0.803 | 0.89 (-3.07,4.85) | 0.660 | -0.29(-4.43,3.85) | 0.889 |
| Correct rate of Stroop |  |  |  |  |  |  |
| 0 serving/week | Reference |  | Reference |  | Reference |  |
| >0-2 servings/week | 0.01(-0.03,0.04) | 0.693 | 0.01(-0.03,0.05) | 0.578 | 0.01(-0.03,0.05) | 0.645 |
| >2 servings/week | 0.02(-0.04,0.07) | 0.530 | 0.03(-0.03,0.09) | 0.340 | 0.02(-0.04,0.08) | 0.496 |
| SI |  |  |  |  |  |  |
| 0 serving/week | Reference |  | Reference |  | Reference |  |
| >0-2 servings/week | -51.94(-174.12,70.23) | 0.405 | -69.67(-197.50,58.17) | 0.285 | -70.04(-201.11,61.02) | 0.295 |
| >2 servings/week | -133.75(-320.10,52.60) | 0.160 | -135.96(-334.22,62.30) | 0.179 | -126.74(-331.61,78.14) | 0.225 |
| WMI |  |  |  |  |  |  |
| 0 serving/week | Reference |  | Reference |  | Reference |  |
| >0-2 servings/week | -2.19(-5.84,1.46) | 0.240 | -2.15(-5.85,1.55) | 0.254 | -1.85(-5.63,1.93) | 0.337 |
| >2 servings/week | -3.29(-8.79,2.21) | 0.241 | -3.67(-9.34,2.00) | 0.204 | -3.61(-9.57,2.35) | 0.235 |

**Abbreviation:** SSB, sugar-sweetened beverages; GEC, Global Executive Component; BRI, Behavioral Regulation Index; MI, Metacognition Index; SI, Stroop interference; WMI, Working Memory Index

^a^ Crude model: without adjustment;

^β^ Adjust model 1: adjusted for age, sex; maternal education; paternal education; family income;

^γ^ Adjust model 2, further adjusted for screen time, physical activity, BMI category and water;

**
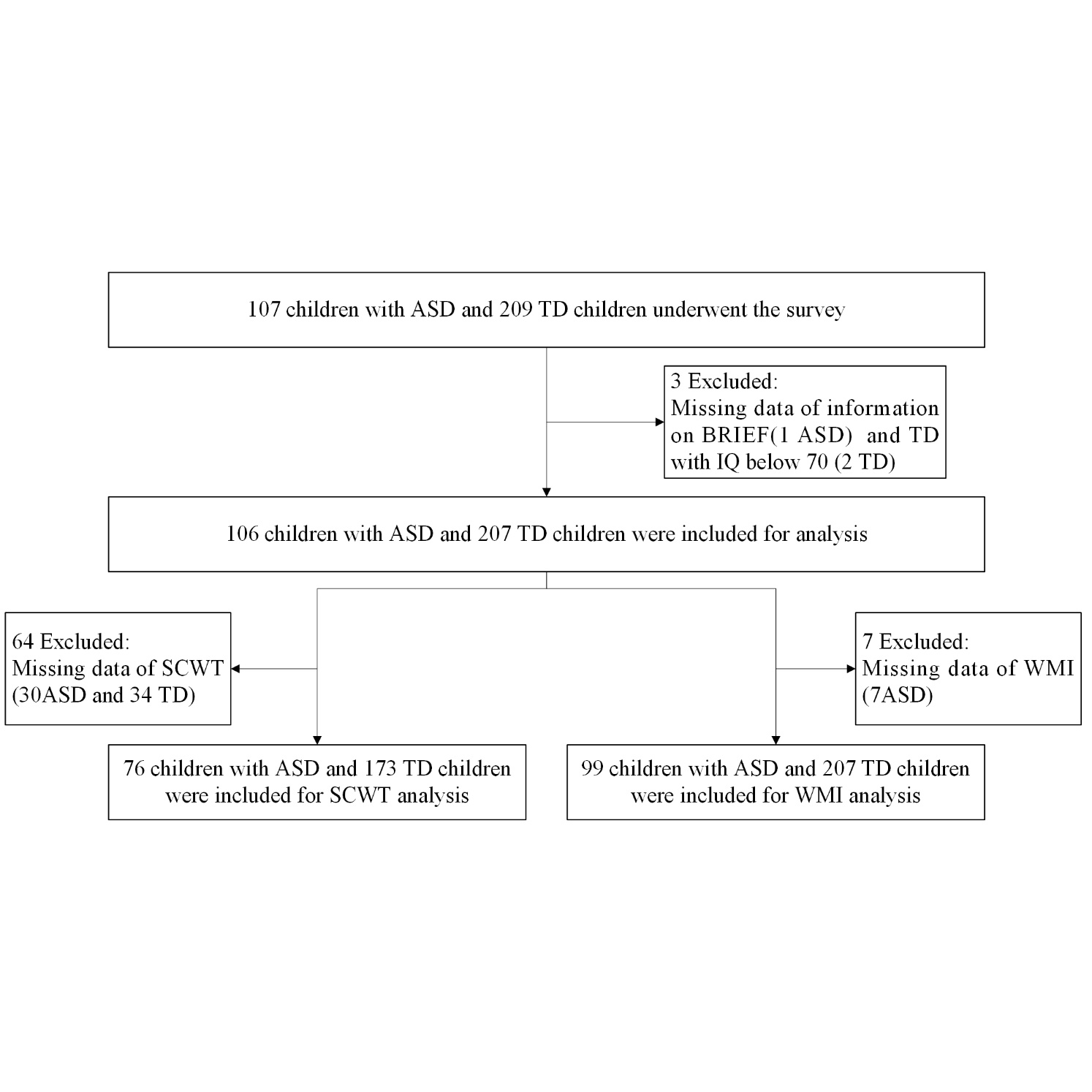
**

**Supplementary Figure S1.** Flowchart of participant inclusion

**Abbreviation:** ASD, autism spectrum disorder; TD, typically developing; BRIEF, Behavior Rating Inventory of Executive Function; IQ, Intelligence Quotient; SCWT, Stroop Color- Word Test; WMI, Working Memory Index.
